# Hypertension Management in Emergency Departments

**DOI:** 10.1093/ajh/hpaa068

**Published:** 2020-04-20

**Authors:** Joseph Miller, Candace McNaughton, Katherine Joyce, Sophia Binz, Phillip Levy

**Affiliations:** 1 Henry Ford Hospital, Detroit, Michigan, USA; 2 Wayne State University, Detroit, Michigan, USA; 3 Vanderbilt University Medical Center and Tennessee Valley Healthcare System VA, Nashville, Tennessee, USA

**Keywords:** blood pressure, emergency department, health disparities, hypertension, hypertensive emergency

## Abstract

**BACKGROUND:**

Elevated blood pressure (BP) is pervasive among patients that visit emergency departments (EDs) for their care.

**METHODS:**

In this review article, we outline the current approach to the management of these individuals and highlight the crucial role emergency medicine clinicians play in reducing the morbidity associated with elevated BP.

**RESULTS:**

We highlight the critical importance of immediate treatment when elevated BP contributes to new or worsening end-organ injury but emphasize that such hypertensive emergencies are rare. For the vast majority of patients with elevated BP in the ED who do not have new or worsening end-organ injury from elevated BP, immediate BP reduction within the ED is not recommended or safe. Nonetheless, within weeks after an ED visit, there is a pressing need to improve the care of patients with elevated or previously undiagnosed hypertension. For many, it may be their only regular point of engagement with the healthcare system. To address this, we present novel perspectives that envision a new role for emergency medicine in chronic hypertension management—one that acknowledges the significant population-level gaps in BP control that contribute to disparities in cardiovascular disease and sets the stage for future changes in systems-based practice.

**CONCLUSIONS:**

Emergency medicine plays a key and evolving role in reducing morbidity associated with elevated BP.

To improve the care of patients with elevated blood pressure (BP) in the emergency department (ED), we provide an overview of the management of hypertensive emergencies, followed by an overview of the management of markedly elevated BP without evidence of end-organ injury. Of equal, if not greater importance, we then deliberate on assessment of patients with nonmarkedly elevated BP and the evolving, essential role that emergency medicine has in population-level hypertension management and in reducing long-term cardiovascular disparities that remain particularly pervasive in urban environments where poor BP control is common. We highlight our experience in Detroit, where hypertension directly contributes to a 1.5-fold increase in years of potential life lost due to heart disease in African Americans compared with non-Hispanic Whites under age 75 years.^1^ We emphasize that reducing such disparities will require the entire house of medicine to work together to overcome the impact that elevated hypertension has on communities nationwide.

An estimated 33–50% of adults in the United States have hypertension, and approximately 41–50% of these adults do not have adequate BP control.^2–4^ More than 145 million ED visits take place each year in the United States, and the estimated prevalence of elevated BP among these patients is close to 45%.^5^ In an analysis of 7 years of data from the Nationwide ED Sample (2006–2012), 165.9 million hypertension-related visits (23.6% of all visits) occurred. During this same period, patients hospitalized declined while hypertension-related ED visits increased.^6^ Data from 2016 showed that approximately 1.2 million ED visits had a chief complaint of essential hypertension.^7^

Based on their training, emergency medicine clinicians naturally focus on the identification and management of hypertensive emergencies for those patients with elevated BP. These critical conditions require rapid evaluation and treatment; however, such patients are rare overall, accounting for <2% of ED visits where high BP is noted.^8,9^ Accordingly, nearly all patients that emergency clinicians see in the ED with elevated BP, including markedly elevated BP (i.e., ≥180/110 mm Hg), are not experiencing an emergency that requires immediate intervention. Despite this, there is often an expectation on the part of patients and other providers, who send their patients to the ED based on a perceived acute risk associated with markedly elevated BP, that something will (or should) be done. Such expectations lead to wide variation in practice patterns.

Furthermore, existing guidelines provide few recommendations as to the management of those with markedly elevated BP absent a hypertensive emergency, leading to questions on who warrants diagnostic testing to look for end-organ injury, how much acute pain or anxiety may contribute to BP in the ED, when initiation or titration of antihypertensive medications is indicated, or how to manage patients with no reliable outpatient follow-up. Emergency clinicians continue to use outdated and incorrect diagnoses such as “hypertension urgency,” a term that implies the need for “something” to be done in the ED setting, despite lack of sound basis in the literature, including current international guidelines.^10^

## ASSESSING AND TREATING HYPERTENSIVE EMERGENCIES

Hypertensive emergencies classically occur in patients with systolic BP (SBP) >220 mm Hg and/or diastolic BP (DBP) >120 mm Hg.^8,11,12^ Nevertheless, lower thresholds can be associated with hypertensive emergencies in the setting of rapid elevations from low-to-normal baseline BP. Furthermore, BP elevations >170/100 mm Hg can cause worsening target-organ injury in select patients. Elevated BP values in isolation, no matter how high they may be, do not by themselves define a hypertensive emergency unless the patient has concomitant acute organ injury for which immediate BP lowering will modify this injury. Thus, terms such as “hypertensive crisis,” which have been historically assigned to all patients with markedly elevated BP have little utility in contemporary practice. New or worsening end-organ injury occurs in the cerebrovascular, cardiovascular, ophthalmologic, hematologic, and renovascular systems.^9,13^ The most common hypertensive emergencies are stroke (ischemic and hemorrhagic) and acute heart failure leading to pulmonary edema. Hypertensive encephalopathy is a rare and poorly understood condition that may reflect direct adverse acute effects of markedly elevated BP on the brain.

Most ED patients with SBP ≥180 mm Hg or DBP ≥110 mm Hg have elevated BP without evidence of end-organ injury. These patients have no immediate indication for rapid BP lowering. Though a common concern among ED providers, hypertensive emergency is rare in patients with elevated BP in the ED.^14^ Thus, while all hypertensive emergencies should be managed with intravenous (IV) antihypertensive therapy to achieve immediate BP reduction, few patients require such intervention. Beyond hypertensive emergency, IV antihypertensive therapy is only indicated for select patients with strict oral medication restrictions and patients abruptly withdrawing from beta-blockade or sympatholytic therapy. These latter patients may benefit from IV labetalol. Given the aforementioned variability in clinical practice, it is not surprising that the majority of IV medications given to achieve immediate BP reduction in the ED are done so inappropriately to patients without new or worsening end-organ injury that can be modified by rapid treatment.^15^

While specific symptoms such as acute dyspnea associated with hypertensive heart failure or chest pain concerning for an acute aortic syndrome may prompt immediate treatment before a full diagnostic evaluation, symptoms alone do not define hypertensive emergencies, and ongoing IV antihypertensive treatment should depend on additional diagnostic tests that confirm acute organ injury. For the large majority of ED patients, presenting clinical features are too nonspecific to prompt immediate IV antihypertensive therapy without confirmatory testing. Table 1 describes common symptoms that emergency medicine clinicians encounter when considering new or worsening end-organ injury in the setting of markedly elevated BP.^8^ For time-sensitive conditions such as acute ischemic stroke, rapid BP lowering may be indicated when BP exceeds 185/110 mm Hg and thrombolytic or endovascular treatment is planned.^16^ Most acute ischemic stroke patients, however, are not candidates for thrombolytic or endovascular therapy, and BP lowering should be avoided. The ischemic penumbra lacks autoregulation of cerebral blood flow and is dependent on systemic perfusion pressure such that acute lowering may worsen ischemia.

**Table 1.** Historical and physical findings associated with hypertensive emergencies

Finding	Diagnostic consideration
Focal neurologic symptoms	Ischemic or hemorrhagic stroke
Fresh flame hemorrhages, papilledema, delirium	Hypertensive encephalopathy
Acute chest pain, back pain	Aortic dissection, myocardial infarction
Acute dyspnea	Pulmonary edema
Seizures, pregnancy	Eclampsia
Hematuria	Acute hypertensive nephrosclerosis
Headache, palpitations, sweating	Pheochromocytoma

**Figure 1.**
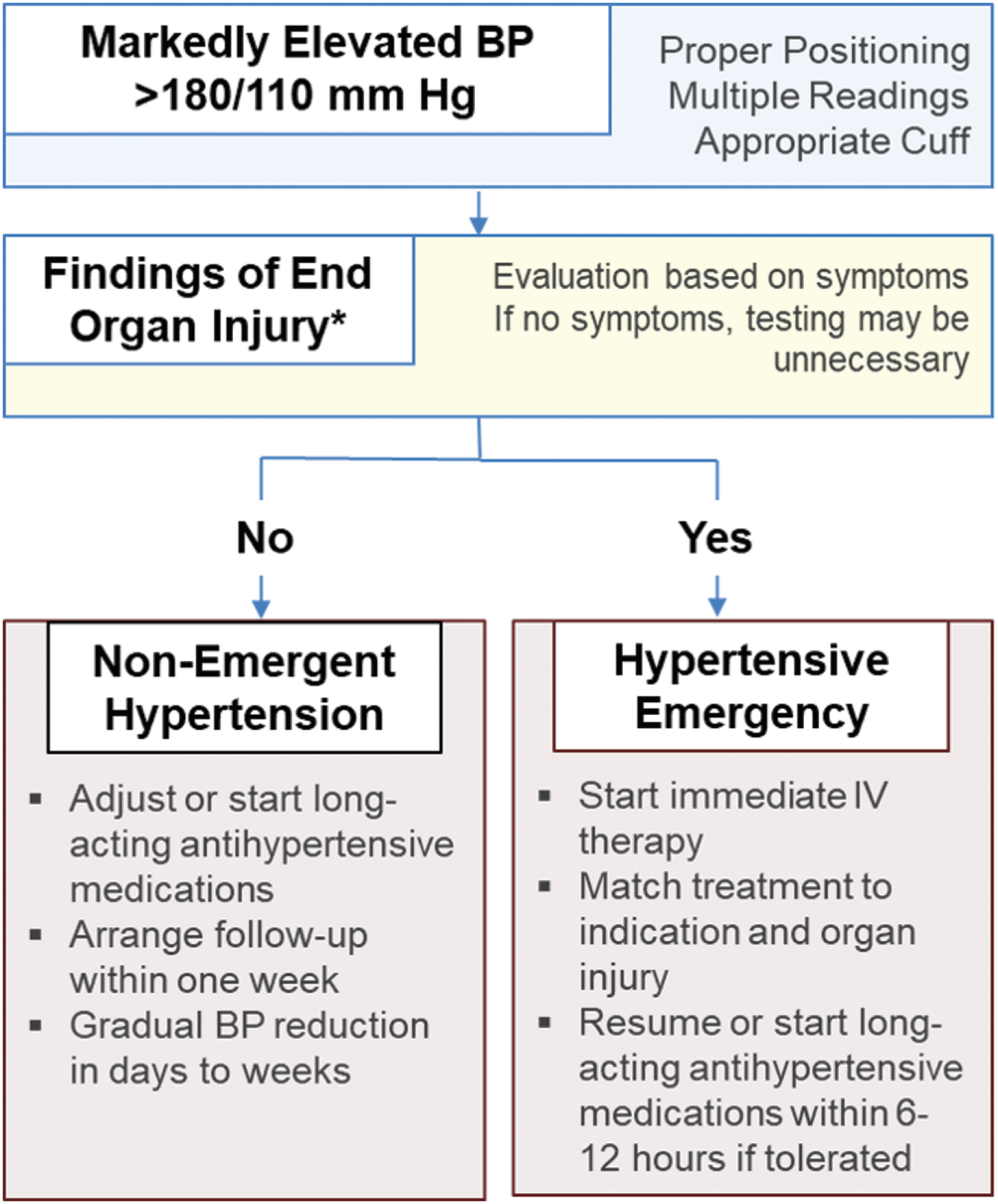
Approach to markedly elevated blood pressure in the emergency department.

Specific tests are indicated for all patients with suspected hypertensive emergency including a basic metabolic profile, complete blood count, urinalysis, electrocardiogram, and chest x-ray. Further workup of patients with markedly elevated BP should be symptom-based and aligned with each associated condition’s differential diagnosis. Figure 1 demonstrates a general approach to patients with markedly elevated BP. In a patient with altered mental status and BP >220/120 mm Hg, the evaluation includes brain imaging by computed tomography to assess for intracerebral hemorrhage or hypertensive encephalopathy. If neither hemorrhage on computed tomography nor alternative reasons for altered mental status are present, magnetic resonance imaging may be warranted.^17^ Likewise, biomarkers of cardiac injury (troponin) and stress (natriuretic peptides) should be obtained for patients with concurrent shortness of breath or chest pain, with the addition of computed tomography angiography of the thorax and abdomen when an acute aortic syndrome is suspected.

Among the diagnoses encompassing the broader grouping of hypertensive emergencies, hypertensive encephalopathy represents the purest form of acute vascular injury from markedly elevated BP. In conditions such as intracerebral hemorrhage and aortic dissection, there is a critical need for immediate BP reduction, but their etiologies are not directly related to a loss of vascular autoregulation. In hypertensive encephalopathy, BP exceeds limits of autoregulation and directly injures the vascular endothelium, leading to cerebral vasodilation and retinal injury often accompanied by glomerular injury and thrombotic microangiopathy.^9,17–19^ Blood flow to cerebral, renal, and other vascular beds is tightly autoregulated to maintain constant perfusion,^20^ but this autoregulation becomes overwhelmed at extreme elevations in BP. Each individual’s BP threshold for a loss of autoregulation, however, is dependent on adaption of their vascular beds. In the typical normotensive patient, the brain maintains constant cerebral flow over a mean arterial pressure (MAP) range from 50 to 160 mm Hg.^21^ In patients with chronically elevated BP, the autoregulatory system shifts to the right to accommodate a persistently greater pressure load, leading to a higher set-point, which can far exceed an SBP of 220 mm Hg or MAP of 160 mm Hg. Because of the adaptation to chronically elevated BP, most thresholds and targets for treatment should be tailored to each patient. Published thresholds apply to large populations and are based upon expert opinion.

With the advent of magnetic resonance imaging, a specific subset of hypertensive encephalopathy known as posterior reversible encephalopathy syndrome has emerged as an important diagnosis. While other etiologies for posterior reversible encephalopathy syndrome exist, such as renal disease, immunosuppressive therapy, erythropoietin use, and thrombotic thrombocytopenic purpura, acute, elevated BP is by far the most common cause.^17^ Besides alterations in mental status, these patients often have seizures and visual changes.^22^ Imaging findings, which typically require magnetic resonance imaging to detect, include vasogenic edema in the posterior brain, especially in the occipital–parietal regions.^22,23^ Despite its distinct diagnosis, posterior reversible encephalopathy syndrome and general hypertensive encephalopathy share the same treatment strategy of rapid BP lowering with IV agents.

## GENERAL TREATMENT CONSIDERATIONS

Current treatment for patients with hypertensive emergencies involves rapid BP reduction to reverse new or worsening end-organ injury and preventing further damage. The American College of Cardiology and the American Heart Association Task Force in their 2017 guidelines recommend reduction of the MAP by 25% within the first hour of treatment. This recommendation is based on evidence that the baseline right-shift in the cerebral autoregulation curve with chronic hypertension is understood to reset approximately 25% above the average MAP. However, it is important to remember that with acute BP elevation, an individual with a hypertensive emergency may be able to withstand greater BP drops as they are on the ascendant portion (as opposed to the plateau) of the autoregulation curve. In general, carefully titrated IV antihypertensive medications are the preferred initial treatment approach in order to limit the risk of cerebral hypoperfusion that may be caused reducing BP too quickly. Over the next 2–6 hours, further BP reduction should occur with the goal SBP 160 mm Hg and DBP 100–110 mm Hg.^10,13^ Following the ED management, inpatient BP reduction aims to reach a normal range gradually within 24–48 hours. For individual disease processes, additional guidelines exist to tailor BP management, and the optimal BP goal for immediate intervention may be greater than 25% of MAP. Table 2 reviews such disease-specific treatment goals. The most common medications indicated for treatment are nicardipine, labetalol, clevidipine, and esmolol. While nitroprusside was a mainstay of treatment for decades, antihypertensive medications such as nicardipine and clevidipine demonstrate similar efficacy, are easy to titrate and have no concern for possible cyanide toxicity.^24–27^ Notably, we do not suggest the use of diuretics for emergent BP treatment. The BP-lowering effect of diuretics is unpredictable, and patients with hypertensive emergencies do not routinely have hypervolemia. The existing literature does not provide sufficient evidence to show that any specific IV antihypertensive agent is superior to another, though the dihydropyridine agents (nicardipine and clevidipine), as well as labetalol, are preferred agents in the setting of neurological hypertensive emergencies.^28^

**Table 2.** Hypertensive emergencies blood pressure goals and treatment options

Category		BP goal (mm Hg)	IV treatment options^a^
*Central nervous system*			
Acute ischemic stroke^10,16,29^	Lytic or endovascular candidate	<185/110 prior to treatment <180/105 post treatment	Nicardipine Labetalol Clevidipine
	Noncandidate	<220/110	
Intracerebral hemorrhage^30^		SBP <160	Nicardipine Labetalol Clevidipine
Hypertensive encephalopathy, PRES^22,23^		Rapid MAP reduction of 25%, then gradual over 24 hours	Nicardipine Labetalol Clevidipine Nitroprusside
*Cardiovascular*			
Aortic dissection^31^		SBP <120 and heart rate ≤60 bpm	Esmolol Labetalol Nicardipine Clevidipine Nitroprusside
*Endocrine*			
Pheochromocytoma		Rapid MAP reduction of 25%, then gradual over 24 hours	Phentolamine Clevidipine Nicardipine
*Pregnancy related* ^10,32^			
Eclampsia, HELLP syndrome		BP <160/110	Labetalol Nicardipine Clevidipine

Abbreviations: BP, blood pressure; IV, intravenous; MAP, mean arterial pressure; PRES, posterior reversible encephalopathy syndrome; SBP, systolic blood pressure.

^a^Intravenous medication dosing: nicardipine (5–15 mg/hour), labetalol (10–20 mg bolus every 10 minutes or 0.5–2 mg/minute infusion), clevidipine (1–2 mg/hour, max 16 mg/hour), nitroprusside (0.25–10 mcg/kg/minute), esmolol (250–500 mcg/kg load over 1 minute then 50–300 mcg/kg/minute infusion), and phentolamine (1–15 mg bolus then 1–40 mg/hour infusion).

## NONEMERGENT HYPERTENSION

While hypertensive emergencies are rare, ED encounters with elevated BP are common and represent valuable opportunities to recognize and address chronic hypertension, particularly among difficult-to-reach patient populations. ED visits related to hypertension rose more than 5% annually from 2006 to 2012.^6^ In 2012, just over 1 million ED visits had a primary diagnosis of hypertension, and almost 27 million ED visits were hypertension related.^6^ Thus, appropriate recognition and management of nonemergent BP elevations for patients with and without diagnosed hypertension should be a core function of emergency medicine clinicians.

One of the primary issues that affect the management of nonemergent BP elevations in the ED is uncertainty around measurement accuracy. BP measurements over minutes to hours using appropriate cuff size and patient positioning can provide valuable information regarding BP variability, range, and trajectory, which can supplement clinic and home BP measurements to guide hypertension diagnosis and medication titration. Multiple studies have shown that BP remains elevated after ED discharge for many patients, and even when BP decreases after ED visits, it does not reach normotension.^33–36^ Elevated SBP and DBP in the ED are both risk factors for incident cardiovascular disease, and the risk rises in a step-wise, dose-dependent fashion with increasing ED BP. The number needed to screen to prevent a single cardiovascular event was 151, but that decreases to 71 among patients with ED BP ≥140/90 mm Hg in the ED. ED BP is particularly informative when measured more than an hour after ED arrival and when it remains elevated over repeated measures,^37,38^ but even triage BP provides important information despite the potential for measurement error due to cuff size and patient positioning. Among ED patients discharged with home BP monitors, more than 88% of patients with a single ED triage BP ≥160/100 mm Hg had a mean home BP ≥135/85 mm Hg.^39^ Another 46% of patients with elevated BP in the ED met criteria for hypertension in follow-up based on home BP monitor, and it is notable that ED physician gestalt was less accurate than mean ED BP for elevated post-ED BP.^40^

Multiple studies have found no evidence for a relationship between ED BP and pain or anxiety.^41–44^ Thus, elevated BP in the ED should not be discounted or explained away by false attribution to pain or anxiety. Given that BP variability is a marker of vascular disease,^45,46^ patients with even temporarily elevated BP in the ED may be at increased cardiovascular risk and therefore benefit from future cardiovascular screening. Long delays in achieving BP control increase the risk of a major adverse cardiovascular event and death,^47^ and younger patients with less elevated BP stand to gain the most benefit from antihypertensive therapy.^48^

### Management consideration

There are no evidence-based thresholds at which asymptomatic but markedly elevated BP in the ED benefits from immediate reduction. While it is important to recognize elevated BP in the ED, rapid BP reduction can cause significant harm by impairing cerebral blood flow, and it has not been shown to improve clinical outcomes except in hypertensive emergencies.^49–51^ Therefore, instead of focusing on immediate BP reduction to “treat numbers,” the goals of ED care for asymptomatic elevated BP are to: (i) assess for new or worsening end-organ injury and confirm lack of hypertensive emergency,^52^ (ii) evaluate risk for persistently elevated BP after ED discharge (which is more likely in patients with persistently elevated BP over repeated measures performed using appropriate cuff size and patient positioning) with consideration of antihypertensive medication prescriptions for those unlikely to successfully follow-up, particularly patients without an established primary care relationship, and (iii) communicate findings with patients and, for those with an existing primary care relationship, with their clinicians with a goal of assuring close outpatient follow-up for repeat BP measurement and possible medication adjustment. Though somewhat more controversial given the nature of the specialty, emergency medicine clinicians may consider briefly addressing lifestyle changes, diet, and exercise, along with medication titration or new initiation of BP-lowering therapy as appropriate.

Whether or not an assessment for end-organ injury should be pursued is also a matter of controversy. This can be accomplished in most instances by history and physical exam. Fundoscopic examination is an important component of the physical exam to detect pressure-related target-organ injury such as papilledema, cotton wool spots, hemorrhages, and exudate. Detection and documentation of less severe but established hypertensive retinopathy confirms that BP is likely chronically elevated, which is important information to share with patients. When uncertainty regarding target-organ injury remains, laboratory testing, electrocardiogram, and/or chest x-ray can be helpful.^53^ However, given the lack of any evidence that additional testing improves diagnostic accuracy beyond history and physical exam, performing such testing routinely in the ED testing is not recommended other than to facilitate other aspects of clinical care, especially new medication initiation.^54^

BP medications can be safely started or titrated in the ED, although there are currently no ED-specific guidelines.^10,55^ Therefore, recommendations for patients in clinic are generalized to the ED, with an emphasis on close outpatient follow-up, need for BP reassessment in 1–2 weeks by a primary care provider, and need to assess electrolytes in 1–2 weeks for patients prescribed an angiotensin-converting enzyme inhibitor or thiazide diuretic. There are no guidelines regarding optimal prescription duration in the ED setting. As in clinic settings, ED treatment should include guidance on lifestyle and medication adherence as well as risks, benefits, side effects, dosing frequency, and anticipated costs. First-line agents include thiazide diuretics, angiotensin-converting enzyme inhibitors, and calcium antagonists.^10^ For ED prescribing, dihydropyridine calcium antagonists are the safer calcium antagonists to prescribe, particularly in older patients and those with severe hypertension that may have undiagnosed ventricular dysfunction. Dihydropyridine calcium antagonists also are advantageous as they can be used with beta-blockers, whereas nondihydropyridine calcium antagonists should not be used. Other important prescribing considerations include patient age, sex, comorbid conditions, current medications, and preferences regarding side effect profiles. For example, older patients with clinical signs of edema or increased intravascular volume may benefit most from a diuretic, and evidence suggests that young Caucasian patients may respond best to an angiotensin-converting enzyme inhibitor as monotherapy.^56^

Another important consideration is race in antihypertensive medication selection. African American patients have higher risk of treatment resistant hypertension and are more likely to require more agents for adequate control. To this end, current guidelines promote the use of dual drug antihypertensive drug therapy.^10^ Recommended treatment is a combination drug therapy with an angiotensin-converting enzyme inhibitor or angiotensin II receptor blocker combined with a calcium channel blocker or thiazide diuretic.^57,58^

Our recommendations are consistent with the 2013 American College of Emergency Physicians’ (ACEP) clinical policy which, based on expert opinion and panel consensus, states that in order to gradually lower BP and/or facilitate chronic BP management, “emergency physicians may choose to initiate hypertension treatment for markedly elevated BP” (defined as SBP ≥180 mm Hg and/or DBP ≥110 mm Hg based on expert opinion). The 2017 ACC/AHA guidelines similarly note that prompt management of very high BP is important to limit the risk of target-organ damage.^10^ Additionally, clinicians should consider factors such as poor primary care follow-up, geriatric patients, or patients at greater risk for adverse outcomes from elevated hypertension such as African American patients in making ED prescribing decisions. Prescribing antihypertensive medication in the ED setting is effective and can be done safely.^55^ Additional resources include mobile health follow-up and engagement, home BP monitor, and community health workers.^40,59–61^

The 2017 ACC/AHA guidelines revising the diagnosis threshold for hypertension and goal BP to 130/80 mm Hg among high-risk patients^10^ adds to the importance of identifying and addressing elevated BP in the ED, particularly for ED patients without access other sources of care.^62^ While there is considerable evidence linked to long-term outcomes that guide non-ED assessment and management of nonemergent BP elevations, evidence to inform ED care is still evolving. The 2013 ACEP clinical policy writing group articulated this noting significant gaps in evidence regarding ED hypertension screening, management of nonemergent elevated BP, antihypertensive medication initiation or titration, and timing of follow-up to improve short- and long-term outcomes while minimizing risks of adverse events.

While the ACC/AHA guidelines have traction in the United States, the 2018 European Society of Cardiology (ESC)/European Society of Hypertension (ESH) guidelines have noted differences in defining hypertension and targets for treatment. The ESC/ESH guidelines define hypertension as ≥140/90 mm Hg and target treatment based on age and risk.^63^ For patients with BP 130–139/85–89 mm Hg, ESC/ESH guidelines recommend lifestyle change and consideration of drug treatment, particularly if patients have high cardiovascular risk. For patients ≤80 years who have BP ≥140/90 mm Hg, drug treatment is indicated. For patients >80 years, ESC/ESH guidelines recommend drug treatment for SBP ≥160 mm Hg. If patients tolerate treatment to maintain BP <140/90 mm Hg, further lowering targets a DBP <80 mm Hg and SBP based on age (SBP target 120–129 mm Hg if age <65 years, 130–139 mm Hg if 65–79 years, and 130–139 mm Hg as tolerated in those >80 years). For patients with known coronary artery disease, therapy is targeted to maintain BP <130/80 mm Hg.^63^ Both guidelines have similar recommendations for BP measurement, lifestyle modifications, and recommended medications. Both recommend strong consideration for single-pill combination antihypertensive medications.^64^

## FUTURE DIRECTIONS

### Population health

As outpatient visits for primary care continue to decline, and hypertension-related ED visits continue to rise,^6,65^ the role of ED care in hypertension and other chronic conditions EDs must evolve to meet these needs.^66^ More than individual patient care, the ED can play an important role in population health through identification of undiagnosed hypertension and recognition of uncontrolled hypertension, particularly for patients without reliable access to primary care, many of whom are underinsured or racial minorities.^62^ Hypertension affects more than half of all non-Hispanic black adults in the United States, with greater severity and earlier onset than in other populations.^58,67^ Hypertension is the most important risk factor for cardiovascular disease among non-Hispanic black adults in the United States, with a more than 30% population attributable risk for cardiovascular disease and 69% population attributable risk for stroke for those less than 60 years of age.^53^ In this age group, a patient’s stroke risk triples for each 10 mm Hg increase in SBP.^53^ Because every patient who presents to the ED has a BP obtained, there is significant opportunity to intercede and reduce risk. However, nothing exists in isolation, and to be effective, hypertension-related initiatives in the ED must be part of a bigger, systems-based effort aimed at improving outcomes. Much like the success of human immunodeficiency syndrome screening efforts from the ED, one can easily envision population health programs that seek to capture patients with elevated or undiagnosed hypertension in the ED and ensure effective linkage to care through directed referral and set appointments. With the support of the Centers for Disease Control and Prevention, such efforts are already underway in EDs located in Detroit and involve, among other things, community health workers who serve to engage patients and provide care continuity beyond the index encounter.

### Personalized treatment

As mentioned, initiation and titration of antihypertensive therapy can be done from the ED, but there is reluctance to do so on the part of emergency medicine clinicians. Yet, this is a relatively simple and potentially highly effective strategy that ED clinicians can use to improve the BP control of their patients. It is also a simple strategy to overcome clinical inertia or the “bystander effect.” The 2017 ACC/AHA guidelines recommend maximizing the dose of antihypertensive medication and then adding addition medication.^10^ Understanding the anticipated BP-lowering effect using tools like the therapeutic intensity score can be used to give providers feedback regarding their prescribing practices and help guide emergency medicine clinicians who seek to do more than simply refer patients on to the next station of care.^68^ The therapeutic intensity score provides a quantifiable estimate of the anticipated impact of antihypertensive therapy, particularly in the setting of multiple antihypertensive medications.

With the steady increase in ED visits and the high proportion of patients with elevated BP, the opportunity for recognition and interventions is shifting from the realm of outpatient medicine and becoming incorporated into the practice of emergency medicine. These opportunities require emergency medicine physicians to be familiar with the definitions of hypertension, the distinction between nonemergent hypertension and hypertensive emergencies, and the treatment for all BP elevations. The ED can serve as a critical partner in systems-level efforts to reduce the morbidity associated with poor hypertension control, particularly in underserved communities.

## References

[1] Michigan Department of Health and Human Services. Michigan Community Health Information http://www.michigan.gov/mdch/0,4612,7-132-2944_5326---,00.html. 2011 Accessed 22 February 2020.

[2] Benjamin EJ, Virani SS, Callaway CW, Chamberlain AM, Chang AR, Cheng S, Chiuve SE, Cushman M, Delling FN, Deo R Heart disease and stroke statistics—2018 update: a report from the American Heart Association. Circulation 2018; 137:e67. 29386200 10.1161/CIR.0000000000000558

[3] Fryar CD, Ostchega Y, Hales CM, Zhang G, Kruszon-Moran D Hypertension prevalence and control among adults: United States, 2015–2016. NCHS Data Brief 2017; 289:1–8. 29155682

[4] Center for Disease Control and Prevention. Vital signs: prevalence, treatment, and control of hypertension—United States, 1999–2002 and 2005–2008. MMWR 2011; 60:103. 21293325

[5] Niska RW Blood pressure measurements at emergency department visits by adults: United States, 2007–2008. NCHS Data Brief 2011; 25:18–24. 22617045

[6] McNaughton CD, Self WH, Zhu Y, Janke AT, Storrow AB, Levy P Incidence of hypertension-related emergency department visits in the United States, 2006 to 2012. Am J Cardiol 2015; 116:1717–1723. 26454813 10.1016/j.amjcard.2015.09.007 PMC4648677

[7] Rui PKK, Ashman JJ Centers for Disease Control and Prevention. National Hospital Ambulatory Medical Care Survey: 2016 Emergency Department Summary Tables. https://www.cdc.gov/nchs/data/nhamcs/web_tables/2016_ed_web_tables.pdf. Accessed 24 February 2020.

[8] Johnson W, Nguyen ML, Patel R Hypertension crisis in the emergency department. Cardiol Clin 2012; 30:533–543. 23102030 10.1016/j.ccl.2012.07.011

[9] Adebayo O, Rogers RL Hypertensive emergencies in the emergency department. Emerg Med Clin North Am 2015; 33:539–551. 26226865 10.1016/j.emc.2015.04.005

[10] Whelton PK, Carey RM, Aronow WS, Casey DE Jr, Collins KJ, Dennison Himmelfarb C, DePalma SM, Gidding S, Jamerson KA, Jones DW, MacLaughlin EJ, Muntner P, Ovbiagele B, Smith SC Jr, Spencer CC, Stafford RS, Taler SJ, Thomas RJ, Williams KA Sr, Williamson JD, Wright JT Jr 2017 ACC/AHA/AAPA/ABC/ACPM/AGS/APhA/ASH/ASPC/NMA/PCNA guideline for the prevention, detection, evaluation, and management of high blood pressure in adults: executive summary: a report of the American College of Cardiology/American Heart Association Task Force on clinical practice guidelines. Circulation 2018; 138:e426–e483. 30354655 10.1161/CIR.0000000000000597

[11] Muiesan ML, Salvetti M, Amadoro V, Di Somma S, Perlini S, Semplicini A, Borghi C, Volpe M, Saba PS, Cameli M An update on hypertensive emergencies and urgencies. J Cardiovasc Med 2015; 16:372–382. 10.2459/JCM.0000000000000223 25575271

[12] Chobanian AV, Bakris GL, Black HR, Cushman WC, Green LA, Izzo JL Jr, Jones DW, Materson BJ, Oparil S, Wright JT Jr Seventh report of the joint national committee on prevention, detection, evaluation, and treatment of high blood pressure. Hypertension 2003; 42:1206–1252. 14656957 10.1161/01.HYP.0000107251.49515.c2

[13] The Seventh Report of the Joint National Committee on Prevention, Detection, Evaluation, and Treatment of High Blood Pressure. National Heart, Lung, and Blood Institute: Bethesda, MD, 2004. 20821851

[14] Deshmukh A, Kumar G, Kumar N, Nanchal R, Gobal F, Sakhuja A, Mehta JL Effect of Joint National Committee VII report on hospitalizations for hypertensive emergencies in the United States. Am J Cardiol 2011; 108:1277–1282. 21890093 10.1016/j.amjcard.2011.06.046

[15] Miller JB, Arter A, Wilson SS, Janke AT, Brody A, Reed BP, Levy PD Appropriateness of bolus antihypertensive therapy for elevated blood pressure in the emergency department. West J Emerg Med 2017; 18:957–962. 28874950 10.5811/westjem.2017.5.33410 PMC5576634

[16] Powers WJ, Rabinstein AA, Ackerson T, Adeoye OM, Bambakidis NC, Becker K, Biller J, Brown M, Demaerschalk BM, Hoh B, Jauch EC, Kidwell CS, Leslie-Mazwi TM, Ovbiagele B, Scott PA, Sheth KN, Southerland AM, Summers DV, Tirschwell DL; American Heart Association Stroke Council 2018 guidelines for the early management of patients with acute ischemic stroke: a guideline for healthcare professionals from the American Heart Association/American Stroke Association. Stroke 2018; 49:e46–e110. 29367334 10.1161/STR.0000000000000158

[17] Miller JB, Suchdev K, Jayaprakash N, Hrabec D, Sood A, Sharma S, Levy PD New developments in hypertensive encephalopathy. Curr Hypertens Rep 2018; 20:13. 29480370 10.1007/s11906-018-0813-y

[18] Akimoto T, Muto S, Ito C, Takahashi H, Takeda S, Ando Y, Kusano E Clinical features of malignant hypertension with thrombotic microangiopathy. Clin Exp Hypertens 2011; 33:77–83. 21214403 10.3109/10641963.2010.503303

[19] Brathwaite L, Reif M Hypertensive emergencies: a review of common presentations and treatment options. Cardiol Clin 2019; 37:275–286. 31279421 10.1016/j.ccl.2019.04.003

[20] Rasulo F, Matta B, Varanini N Cerebral blood flow monitoring. In Prabhakar H (ed), Neuromonitoring Techniques. Elsevier: London, 2018.

[21] Paulson OB, Waldemar G, Schmidt JF, Strandgaard S Cerebral circulation under normal and pathologic conditions. Am J Cardiol 1989; 63:2C–5C. 10.1016/0002-9149(89)90396-2 2643850

[22] Fischer M, Schmutzhard E Posterior reversible encephalopathy syndrome. J Neurol 2017; 264:1608–1616. 28054130 10.1007/s00415-016-8377-8 PMC5533845

[23] Servillo G, Bifulco F, De Robertis E, Piazza O, Striano P, Tortora F, Striano S, Tufano R Posterior reversible encephalopathy syndrome in intensive care medicine. Intensive Care Med 2007; 33:230–236. 17119920 10.1007/s00134-006-0459-0

[24] Udeh CI, Ting M, Arango M, Mick S Delayed presentation of nitroprusside-induced cyanide toxicity. Ann Thorac Surg 2015; 99:1432–1434. 25841829 10.1016/j.athoracsur.2014.05.097

[25] Miller JB, Kinni H, Amer A, Levy PD Therapies to reduce blood pressure acutely. Curr Hypertens Rep 2016; 18:43. 27125389 10.1007/s11906-016-0651-8

[26] Manning L, Robinson TG, Anderson CS Control of blood pressure in hypertensive neurological emergencies. Curr Hypertens Rep 2014; 16:436. 24771058 10.1007/s11906-014-0436-x

[27] Ipek E, Oktay AA, Krim SR Hypertensive crisis: an update on clinical approach and management. Curr Opin Cardiol 2017; 32:397–406. 28306673 10.1097/HCO.0000000000000398

[28] Perez MI, Musini VM Pharmacological interventions for hypertensive emergencies. Cochrane Database Syst Rev 2008:CD003653. 18254026 10.1002/14651858.CD003653.pub3 PMC6991936

[29] Adams HP Jr., del Zoppo G, Alberts MJ, Bhatt DL, Brass L, Furlan A, Grubb RL, Higashida RT, Jauch EC, Kidwell C, Lyden PD, Morgenstern LB, Qureshi AI, Rosenwasser RH, Scott PA, Wijdicks EF; American Heart Association; American Stroke Association Stroke Council; Clinical Cardiology Council; Cardiovascular Radiology and Intervention Council; Atherosclerotic Peripheral Vascular Disease and Quality of Care Outcomes in Research Interdisciplinary Working Groups. Guidelines for the early management of adults with ischemic stroke: a guideline from the American Heart Association/American Stroke Association Stroke Council, Clinical Cardiology Council, Cardiovascular Radiology and Intervention Council, and the Atherosclerotic Peripheral Vascular Disease and Quality of Care Outcomes in Research Interdisciplinary Working Groups: the American Academy of Neurology affirms the value of this guideline as an educational tool for neurologists. Stroke 2007; 38:1655–1711. 17431204 10.1161/STROKEAHA.107.181486

[30] Hemphill JC 3rd, Greenberg SM, Anderson CS, Becker K, Bendok BR, Cushman M, Fung GL, Goldstein JN, Macdonald RL, Mitchell PH, Scott PA, Selim MH, Woo D; American Heart Association Stroke Council; Council on Cardiovascular and Stroke Nursing; Council on Clinical Cardiology. Guidelines for the management of spontaneous intracerebral hemorrhage: a guideline for healthcare professionals from the American Heart Association/American Stroke Association. Stroke 2015; 46:2032–2060. 26022637 10.1161/STR.0000000000000069

[31] Hiratzka LF, Bakris GL, Beckman JA, Bersin RM, Carr VF, Casey DE Jr, Eagle KA, Hermann LK, Isselbacher EM, Kazerooni EA, Kouchoukos NT, Lytle BW, Milewicz DM, Reich DL, Sen S, Shinn JA, Svensson LG, Williams DM; American College of Cardiology Foundation; American Heart Association Task Force on Practice Guidelines; American Association for Thoracic Surgery; American College of Radiology; American Stroke Association; Society of Cardiovascular Anesthesiologists; Society for Cardiovascular Angiography and Interventions; Society of Interventional Radiology; Society of Thoracic Surgeons; Society for Vascular Medicine. 2010 ACCF/AHA/AATS/ACR/ASA/SCA/SCAI/SIR/STS/SVM guidelines for the diagnosis and management of patients with thoracic aortic disease: executive summary. A report of the American College of Cardiology Foundation/American Heart Association Task Force on Practice Guidelines, American Association for Thoracic Surgery, American College of Radiology, American Stroke Association, Society of Cardiovascular Anesthesiologists, Society for Cardiovascular Angiography and Interventions, Society of Interventional Radiology, Society of Thoracic Surgeons, and Society for Vascular Medicine. Catheter Cardiovasc Interv 2010; 76:E43–E86. 20687249 10.1002/ccd.22537

[32] Practice CoO. ACOG Committee Opinion No. 767 summary: emergent therapy for acute-onset, severe hypertension during pregnancy and the postpartum period. Obstet Gynecol 2019; 133:409–412. 30681541 10.1097/AOG.0000000000003082

[33] Chernow SM, Iserson KV, Criss E Use of the emergency department for hypertension screening: a prospective study. Ann Emerg Med 1987; 16:180–182. 3800093 10.1016/s0196-0644(87)80012-4

[34] Backer HD, Decker L, Ackerson L Reproducibility of increased blood pressure during an emergency department or urgent care visit. Ann Emerg Med 2003; 41:507–512. 12658251 10.1067/mem.2003.151

[35] Cienki JJ, DeLuca LA Agreement between emergency medical services and expert blood pressure measurements. J Emerg Med 2012; 43:64–68. 21982624 10.1016/j.jemermed.2011.02.018

[36] Cienki JJ, Deluca LA, Feaster DJ Course of untreated high blood pressure in the emergency department. West J Emerg Med 2011; 12:421–425. 22224131 10.5811/westjem.2011.3.1764 PMC3236139

[37] Dieterle T, Schuurmans MM, Strobel W, Battegay EJ, Martina B Moderate-to-severe blood pressure elevation at ED entry: hypertension or normotension? Am J Emerg Med 2005; 23:474–479. 16032614 10.1016/j.ajem.2005.02.046

[38] Oras P, Häbel H, Skoglund PH, Svensson P Elevated blood pressure in the emergency department: a risk factor for incident cardiovascular disease. Hypertension 2020; 75:229–236. 31786971 10.1161/HYPERTENSIONAHA.119.14002

[39] Goldberg EM, Wilson T, Saucier C, Brody AM, Levy PD, Eaton CB, Merchant RC Achieving the BpTRUth: emergency department hypertension screening and the Centers for Medicare & Medicaid Services quality measure. J Am Soc Hypertens 2017; 11:290–294. 28412275 10.1016/j.jash.2017.03.003 PMC6996104

[40] Goldberg EM, Wilson T, Jambhekar B, Marks SJ, Boyajian M, Merchant RC Emergency department-provided home blood pressure devices can help detect undiagnosed hypertension. High Blood Press Cardiovasc Prev 2019; 26:45–53. 30659517 10.1007/s40292-019-00300-0 PMC6407636

[41] Tanabe P, Cline DM, Cienki JJ, Egging D, Lehrmann JF, Baumann BM Barriers to screening and intervention for ED patients at risk for undiagnosed or uncontrolled hypertension. J Emerg Nurs 2011; 37:17–23. 21237363 10.1016/j.jen.2009.11.017

[42] Baumann BM, Cline DM, Cienki JJ, Egging D, Lehrmann JF, Tanabe P Provider self-report and practice: reassessment and referral of emergency department patients with elevated blood pressure. Am J Hypertens 2009; 22:604–610. 19265789 10.1038/ajh.2009.44

[43] Tanabe P, Persell SD, Adams JG, McCormick JC, Martinovich Z, Baker DW Increased blood pressure in the emergency department: pain, anxiety, or undiagnosed hypertension? Ann Emerg Med 2008; 51:221–229. 18207606 10.1016/j.annemergmed.2007.10.017

[44] Tanabe P, Steinmann R, Kippenhan M, Stehman C, Beach C Undiagnosed hypertension in the ED setting—an unrecognized opportunity by emergency nurses. J Emerg Nurs 2004; 30:225–229. 15192674 10.1016/j.jen.2004.01.009

[45] Ohkuma T, Woodward M, Jun M, Muntner P, Hata J, Colagiuri S, Harrap S, Mancia G, Poulter N, Williams B, Rothwell P, Chalmers J; ADVANCE Collaborative Group Prognostic value of variability in systolic blood pressure related to vascular events and premature death in Type 2 diabetes mellitus: the ADVANCE-ON Study. Hypertension 2017; 70:461–468. 28584014 10.1161/HYPERTENSIONAHA.117.09359

[46] Filomena J, Riba-Llena I, Vinyoles E, Tovar JL, Mundet X, Castañé X, Vilar A, López-Rueda A, Jiménez-Baladó J, Cartanyà A, Montaner J, Delgado P; ISSYS Investigators Short-term blood pressure variability relates to the presence of subclinical brain small vessel disease in primary hypertension. Hypertension 2015; 66:634–640; discussion 445. 26101344 10.1161/HYPERTENSIONAHA.115.05440

[47] Xu W, Goldberg SI, Shubina M, Turchin A Optimal systolic blood pressure target, time to intensification, and time to follow-up in treatment of hypertension: population based retrospective cohort study. BMJ 2015; 350:h158. 25655523 10.1136/bmj.h158 PMC4353282

[48] Hermida RC, Crespo JJ, Dominguez-Sardina M, Otero A, Moya A, Rios MT, Sineiro E, Castineira MC, Callejas PA, Pousa L, Salgado JL, Duran C, Sanchez JJ, Fernandez JR, Mojon A, Ayala DE, Hygia Project I. Bedtime hypertension treatment improves cardiovascular risk reduction: the Hygia Chronotherapy Trial. Eur Heart J 2019; e-pub ahead of print. 10.1093/eurheartj/ehz754 31641769

[49] Wachter RM Symptomatic hypotension induced by nifedipine in the acute treatment of severe hypertension. Arch Intern Med 1987; 147:556–558. 3827433

[50] O’Mailia JJ, Sander GE, Giles TD Nifedipine-associated myocardial ischemia or infarction in the treatment of hypertensive urgencies. Ann Intern Med 1987; 107:185–186. 3605898 10.7326/0003-4819-107-2-185

[51] Grossman E, Messerli FH, Grodzicki T, Kowey P Should a moratorium be placed on sublingual nifedipine capsules given for hypertensive emergencies and pseudoemergencies? JAMA 1996; 276:1328–1331. 8861992

[52] Baumann BM, Cline DM, Pimenta E Treatment of hypertension in the emergency department. J Am Soc Hypertens 2011; 5:366–377. 21719370 10.1016/j.jash.2011.05.002

[53] Clark D 3rd, Colantonio LD, Min YI, Hall ME, Zhao H, Mentz RJ, Shimbo D, Ogedegbe G, Howard G, Levitan EB, Jones DW, Correa A, Muntner P Population-attributable risk for cardiovascular disease associated with hypertension in black adults. JAMA Cardiol 2019; 4:1194–1202. 31642869 10.1001/jamacardio.2019.3773 PMC6813577

[54] Wolf SJ, Lo B, Shih RD, Smith MD, Fesmire FM; American College of Emergency Physicians Clinical Policies Committee Clinical policy: critical issues in the evaluation and management of adult patients in the emergency department with asymptomatic elevated blood pressure. Ann Emerg Med 2013; 62:59–68. 23842053 10.1016/j.annemergmed.2013.05.012

[55] Brody A, Rahman T, Reed B, Millis S, Ference B, Flack JM, Levy PD Safety and efficacy of antihypertensive prescription at emergency department discharge. Acad Emerg Med 2015; 22:632–635. 25904073 10.1111/acem.12660 PMC5050391

[56] Dickerson JE, Hingorani AD, Ashby MJ, Palmer CR, Brown MJ Optimisation of antihypertensive treatment by crossover rotation of four major classes. Lancet 1999; 353:2008–2013. 10376615 10.1016/s0140-6736(98)07614-4

[57] Ojji DB, Mayosi B, Francis V, Badri M, Cornelius V, Smythe W, Kramer N, Barasa F, Damasceno A, Dzudie A, Jones E, Mondo C, Ogah O, Ogola E, Sani MU, Shedul GL, Shedul G, Rayner B, Okpechi IG, Sliwa K, Poulter N; CREOLE Study Investigators Comparison of dual therapies for lowering blood pressure in Black Africans. N Engl J Med 2019; 380:2429–2439. 30883050 10.1056/NEJMoa1901113

[58] Flack JM, Sica DA, Bakris G, Brown AL, Ferdinand KC, Grimm RH Jr, Hall WD, Jones WE, Kountz DS, Lea JP, Nasser S, Nesbitt SD, Saunders E, Scisney-Matlock M, Jamerson KA; International Society on Hypertension in Blacks Management of high blood pressure in Blacks: an update of the International Society on Hypertension in Blacks consensus statement. Hypertension 2010; 56:780–800. 20921433 10.1161/HYPERTENSIONAHA.110.152892

[59] Meurer WJ, Dome M, Brown D, Delemos D, Oska S, Gorom V, Skolarus L Feasibility of emergency department-initiated, mobile health blood pressure intervention: an exploratory, randomized clinical trial. Acad Emerg Med 2019; 26:517–527. 30659702 10.1111/acem.13691 PMC6945785

[60] Foster B, Dawood K, Pearson C, Manteuffel J, Levy P Community health workers in the emergency department—can they help with chronic hypertension care. Curr Hypertens Rep 2019; 21:49. 31115736 10.1007/s11906-019-0955-6

[61] Bone LR, Mamon J, Levine DM, Walrath JM, Nanda J, Gurley HT, Noji EK, Ward E Emergency department detection and follow-up of high blood pressure: use and effectiveness of community health workers. Am J Emerg Med 1989; 7:16–20. 2914043 10.1016/0735-6757(89)90077-6

[62] Levy PD Whose job is it anyway? Acad Emerg Med 2019; 26:584–586. 30946513 10.1111/acem.13762

[63] Williams B, Mancia G, Spiering W, Agabiti Rosei E, Azizi M, Burnier M, Clement DL, Coca A, de Simone G, Dominiczak A, Kahan T, Mahfoud F, Redon J, Ruilope L, Zanchetti A, Kerins M, Kjeldsen SE, Kreutz R, Laurent S, Lip GYH, McManus R, Narkiewicz K, Ruschitzka F, Schmieder RE, Shlyakhto E, Tsioufis C, Aboyans V, Desormais I; ESC Scientific Document Group 2018 ESC/ESH Guidelines for the management of arterial hypertension. Eur Heart J 2018; 39:3021–3104. 30165516 10.1093/eurheartj/ehy339

[64] Bakris G, Ali W, Parati G ACC/AHA versus ESC/ESH on hypertension Guidelines: JACC guideline comparison. J Am Coll Cardiol 2019; 73:3018–3026. 31196460 10.1016/j.jacc.2019.03.507

[65] Ganguli I, Lee TH, Mehrotra A Evidence and implications behind a national decline in primary care visits. J Gen Intern Med 2019; 34:2260–2263. 31243711 10.1007/s11606-019-05104-5 PMC6816587

[66] Rosenbaum L The not-my-problem problem. N Engl J Med 2019; 380:881–885. 30811917 10.1056/NEJMms1813431

[67] Muntner P, Carey RM, Gidding S, Jones DW, Taler SJ, Wright JT Jr, Whelton PK Potential US population impact of the 2017 ACC/AHA high blood pressure guideline. Circulation 2018; 137:109–118. 29133599 10.1161/CIRCULATIONAHA.117.032582 PMC5873602

[68] Levy PD, Willock RJ, Burla M, Brody A, Mahn J, Marinica A, Nasser SA, Flack JM Total antihypertensive therapeutic intensity score and its relationship to blood pressure reduction. J Am Soc Hypertens 2016; 10:906–916. 27856202 10.1016/j.jash.2016.10.005 PMC5427634

